# Insufficient Labor Epidural Analgesia Due to Asymptomatic Sacral Perineural Cyst: A Case Report

**DOI:** 10.7759/cureus.74568

**Published:** 2024-11-27

**Authors:** Ryuichi Wakata, Mihou Inoue, Kyomi Kasai

**Affiliations:** 1 Department of Anesthesiology, Adachi Hospital, Kyoto, JPN

**Keywords:** asymptomatic sacral cyst, labor analgesia, labor epidural analgesia, lumbar epidural anesthesia, sacral perineural cyst

## Abstract

Lumbar epidural anesthesia is widely used for labor epidural analgesia (LEA), but it often results in insufficient analgesia in the sacral region. We report a case where we performed LEA using lumbar epidural anesthesia, and an asymptomatic sacral perineural cyst was considered the potential cause of inadequate analgesia in the sacral region. A 33-year-old primigravida was admitted with premature rupture of membranes. Lumbar epidural anesthesia was performed after spontaneous labor occurred. Labor epidural analgesia alleviated the pain, but it worsened again during the second stage of labor. No nerve blockade in the sacral region was confirmed. Despite catheter adjustment and additional medication, pain relief was not achieved. A review of past imaging studies revealed an asymptomatic sacral perineural cyst. A sacral perineural cyst can be considered one of the causes of insufficient analgesia in the sacral region during LEA. We propose strategies for managing similar cases and emphasize the importance of pre-anesthetic imaging review when available.

## Introduction

Labor epidural analgesia (LEA) has gained widespread acceptance as an effective method for labor pain management. In recent years, there has been an increase in popularity in Japan, with 8.6% of people using it in 2020 [1]. With more facilities offering LEA, the focus is now on improving service quality. During the first stage of labor, women typically experience visceral pain due to uterine contractions and cervical dilation [2]. This pain is transmitted to the spinal cord through the Th10 to L1 nerve roots. In the second stage of labor, the predominant type of pain shifts to somatic pain. This arises from the stretching of the perineum and is relayed through the S2 to S4 nerve roots. Consequently, achieving adequate labor analgesia needs extensive neural blockade spanning multiple spinal segments. Lumbar epidural analgesia is used to obtain this broad neural blockade. However, it often provides insufficient coverage in the sacral region. When sacral region analgesia is insufficient, physicians may administer additional medications via the epidural catheter, adjust the catheter, or replace it.

In this report, we present a case where, despite the administration of lumbar epidural analgesia for labor, the desired sacral nerve blockade was not achieved. Upon further investigation through past imaging studies, an asymptomatic cyst was found within the sacral canal, potentially contributing to insufficient analgesia. This unique aspect underscores the variability in patient response to standard analgesia techniques and highlights the importance of considering anatomical anomalies or pathological conditions when managing labor pain.

## Case presentation

A 33-year-old woman, gravida 1 para 0, 170 cm, 73 kg, was admitted to our hospital at 38 weeks and five days of gestation due to premature rupture of the membranes. Her medical history included upper thoracic scoliosis and previous inguinal hernia repair. Spontaneous labor began 13 hours after admission, and the patient requested LEA 18 hours post-admission when cervical dilation was 6 cm.

In the left lateral decubitus position, a 17G Tuohy needle was inserted at the L3/4 intervertebral space, and a loss of resistance was obtained at a depth of 5.5 cm. Subsequently, a 19G multi-orifice epidural catheter was placed at a depth of 5.5 cm. No complications occurred during catheter placement, and aspiration yielded neither blood nor cerebrospinal fluid.

Following an epidural test dose of 3 ml of 1% lidocaine, 100 μg of fentanyl (2 ml), and 9 ml of 0.25% bupivacaine were given in divided doses. Thirty minutes after the epidural administration of bupivacaine and fentanyl, the patient reported a reduction in labor pain to 5/10. The epidural pump was set to deliver intermittent boluses of 6 ml of 0.1% ropivacaine with 2 μg/ml fentanyl every 45 minutes, starting 30 minutes after LEA initiation, with patient-controlled epidural analgesia boluses of 5 ml every five minutes as needed.

One hour after LEA initiation, the cervical dilation was complete. However, perineal pain persisted, and sensory block assessment using temperature sensation revealed a block limited to the lumbar region. Confirming the absence of sacral nerve blockade through temperature sensation, 2 ml of 2% lidocaine was administered 3 hours after LEA initiation, which proved ineffective. Consequently, 30 minutes later, the epidural catheter was withdrawn by 1 cm, and 3 ml of 0.75% ropivacaine was administered, but the pain remained intense.

**Figure 1:**
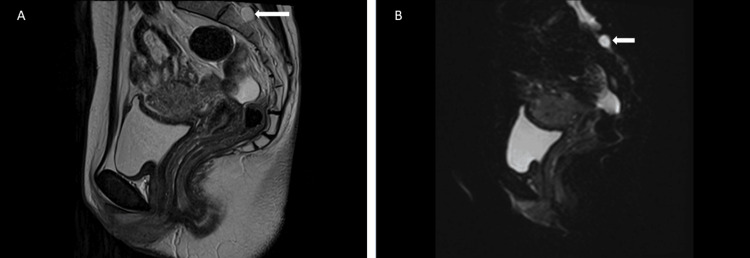
Magnetic resonance imaging of the pelvis Figure 1A: The sagittal T2-weighted image demonstrates a 13mm sacral perineural cyst at the S2 level (white arrow); Figure 1B: The sagittal diffusion-weighted image demonstrates that the signal intensity of the cyst is the same as that of the cerebrospinal fluid (white arrow).

She complained of dysesthesia in her lower limbs, and no additional drugs were administered. She also declined to have the epidural catheter replaced. One hour and a half later, as sacral region analgesia could not be confirmed, an episiotomy was performed under infiltration anesthesia with 1% mepivacaine, and a male infant weighing 3576 g was delivered with the assistance of the Kristeller maneuver. The newborn had Apgar scores of nine and nine after one and five minutes, respectively. The estimated amount of bleeding at delivery was 590 ml, and the total delivery time was 10 hours. The epidural catheter was removed just after delivery. The dysesthesia in the lower limbs disappeared 12 hours after delivery, and no other complications were observed. Upon reviewing the pelvic MRI images taken before inguinal hernia repair, a sacral cyst at S2 was noted (Figure 1), which could be considered a potential cause for the insufficient analgesia in the sacral region.

## Discussion

This case report presents a patient who experienced insufficient LEA due to an asymptomatic sacral perineural cyst, highlighting several important aspects of neuraxial anesthesia in obstetric practice. Sacral perineural cysts, first described by Tarlov in 1938, are incidental findings in 2%-10% of patients undergoing imaging studies, with the most common location being the S1-S2 vertebral level [3-6]. While often asymptomatic, these cysts can significantly impact the efficacy of LEA by interfering with the spread of local anesthetic in the epidural space, altering local anatomy, and changing the volume and pressure dynamics within the epidural space.

The sacral region’s unique anatomical characteristics, including its larger epidural space volume and the thickness of the dural matter surrounding the sacral nerve roots, can already hinder effective nerve blockade [7]. The presence of a sacral perineural cyst further compounds this challenge. Moreover, pregnancy-induced physiological changes can exacerbate the difficulties in achieving adequate sacral analgesia [8]. These changes include increased intra-abdominal pressure, engorgement of epidural veins, and softening of ligaments. As labor progresses, particularly during the second stage, the descending fetus increases stimulation of sacral nerve roots, demanding additional anesthetic coverage. The combination of these factors with an asymptomatic sacral perineural cyst can result in inadequate pain relief, as observed in our case.

When encountering LEA failure, especially with inadequate sacral analgesia, clinicians should consider a broad differential diagnosis, including technical factors (e.g., catheter misplacement or migration), pharmacological factors (e.g., inadequate dosing or drug distribution), patient factors (e.g., anatomical variations, including scoliosis, and pathological conditions, including sacral perineural cysts), and obstetric factors (e.g., malposition of the fetus). Our case emphasizes the importance of including anatomical variations in this differential, particularly when other common causes have been ruled out.

Based on our experience and a review of the literature, we propose a comprehensive approach to managing similar cases. This approach begins with a thorough pre-anesthetic evaluation, including a review of available imaging studies, particularly of the lumbosacral region, to identify potential anatomical variations or abnormalities that might affect LEA efficacy. When encountering LEA failure, a stepwise troubleshooting process should be employed, systematically addressing technical, pharmacological, and patient factors before considering advanced imaging or alternative techniques.

Alternative analgesia techniques such as combined spinal-epidural anesthesia, continuous spinal anesthesia, or pudendal nerve blocks should be considered. However, caution is warranted, as there have been reports of complications with neuraxial anesthesia in patients with sacral perineural cysts [9, 10]. Patient counseling is crucial; individuals with known sacral perineural cysts should be informed about the potential for reduced LEA efficacy, and alternative pain management options should be discussed before labor. In cases of unexplained LEA failure, post-delivery imaging should be considered to investigate potential anatomical causes, which may inform future anesthetic management.

The safety of neuraxial anesthesia in patients with sacral perineural cysts remains a topic of debate. While some studies report successful use of spinal or epidural anesthesia without complications [11, 12], others document serious adverse events such as cauda equina syndrome following combined spinal-epidural anesthesia [9, 10]. This variability in outcomes underscores the need for careful risk-benefit analysis when considering neuraxial techniques in patients with known sacral perineural cysts, enhanced vigilance and monitoring during and after the procedure, and further research to establish evidence-based guidelines for managing these patients.

This case report highlights several areas for future research and clinical practice improvement. Prospective studies are needed to determine the true incidence of LEA failure associated with sacral perineural cysts. The development of screening protocols to identify patients at risk for LEA failure due to anatomical variations would be beneficial. Investigation of optimal anesthetic techniques for patients with known sacral perineural cysts is warranted. Additionally, the exploration of advanced imaging techniques, such as 3D MRI, could better elucidate the impact of sacral perineural cysts on local anesthetic spread in the epidural space.

## Conclusions

In conclusion, this case underscores the importance of considering anatomical variations, such as sacral perineural cysts, in the differential diagnosis of LEA failure, particularly when sacral analgesia is inadequate. While these cysts are often asymptomatic, they can significantly impact the spread of local anesthetics in the epidural space. Anesthesiologists should be aware of this potential complication and consider a structured approach to diagnosis and management when encountering similar cases. Furthermore, this report highlights the value of thorough pre-anesthetic evaluations, including a review of available imaging studies, which may reveal important anatomical information to guide anesthetic management. As our understanding of these challenging cases evolves, we can work towards optimizing pain management strategies for all parturients, ensuring safe and effective analgesia during labor and delivery.
